# Editorial: CBASP in the Treatment of Persistent Depressive Disorder

**DOI:** 10.3389/fpsyt.2021.804602

**Published:** 2021-12-16

**Authors:** Jenneke Wiersma, Philipp Klein, Elisabeth Schramm, Toshi Furukawa, Todd Favorite

**Affiliations:** ^1^Instituut voor Directieve Interventies, Amsterdam, Netherlands; ^2^Department of Psychiatry and Psychotherapy, University Medical Center Schleswig-Holstein Lübeck, Kiel, Germany; ^3^Department of Psychiatry and Psychotherapy, University of Freiburg Medical Center, Freiburg im Breisgau, Germany; ^4^Institute of Research, Kyoto University, Kyoto, Japan; ^5^Department of Psychiatry, Michigan Medicine, University of Michigan, Ann Arbor, MI, United States

**Keywords:** persistent depressive disorder, cognitive behavioral analysis system of psychotherapy, comorbidity, effectiveness, group

This special issue seeks to contribute to a better understanding of patients with a persistent depressive disorder (PDD) and on how to optimize treatment for this group of patients. Starting with a case description:

*John is a 39-year-old man. He is diagnosed with PDD. His depression started by the age of 18. He grew up with a depressed mother, who was in and out of hospital during his childhood. His father had a hard time taking care of him and his siblings and hit them when they did not obey or if they said something he did not like. John learned to stay out of his parents' way and to take care of himself. He is currently working as an accountant in a small firm. He met his wife at the age of 23 and married her straight away. They have a son, now at the age of 15 years, hitting puberty. John has trouble handling him. His depression got worse in the last couple of months and he seeks treatment. During treatment it becomes clear that it is difficult for John to assert himself. He is afraid to get punished or rejected when he expresses something negative. In turn, he withdraws when these situations appear*.

John is referred to a Cognitive Behavioral Analysis System of Psychotherapy (CBASP) therapist since CBASP is recommended as first line psychotherapeutic treatment for persistent depressive disorder by several national and international treatment guidelines (e.g., the Danish, German, Canadian guidelines and the European Psychiatric Association). CBASP is developed by James McCullough Jr. who is stating in his contribution for this special issue: “Becoming a successful CBASP psychotherapist is not a simple undertaking.” Patients with PDD, like John, are difficult to treat because of the entrenched cognitive-emotional-behavioral patterns they bring to treatment, exhibited in overlearned interpersonal fear and avoidance (“it is better to stay away from others”) often due to abusive developmental histories (in John's case a depressed mother and an aggressive father).

McCullough states that these toxic developmental histories derail normal social-emotional maturational development and entrap patients in a preoperational state of functioning. The term “preoperational” stems from Piaget's theory of cognitive development in children. In this preoperational state, patients are not connected to their environment (John withdraws whenever something negatives happens). CBASP practitioners are required to actualize a personal relationship with their patients that seeks to modify this preoperational state of being, this technique is labeled *Disciplined Personal Involvement*. Disciplined Personal Involvement addresses the interpersonal fear and avoidance and teaches patients to connect with others, starting with the therapist.

Like John, many patients grow up with depressed mothers (or fathers). A study on the effects of maternal depression, and more specifically, maternal chronic depression, on offspring's risk for depression, anxiety, and externalizing symptoms, by Silver et al., state that given the potential long-term effects of maternal chronic depression on offspring, early identification, appropriate treatment, and follow-up of depressed women and their children should be key priority.

As our patient John grew up with a depressed mother and an aggressive father both not able to handle and take care of their children, John learned to avoid his parents and disconnect with his environment, which kept him in a preoperational state; thinking “whatever I do, nothing will ever change.” Sondermann et al. examined the influence of preoperational thinking on depressive symptom severity over 2 years. They found that higher levels of preoperational thinking are associated with higher depressive symptom severity over time. Their results confirm the assumption of CBASP that preoperational thinking is an important factor contributing to the maintenance of depression and therefore needs to be addressed in psychotherapy.

Most PDD patients feel isolated and lonely. Nenov-Matt et al. contribution focusses on the importance of loneliness, since loneliness has been associated with the development of mental disorders and chronic illness trajectories. In a cross-diagnostic study loneliness was examined by comparing PDD and Borderline Personality Disorder (BPD) patients with healthy controls in its interplay with symptom burden, social network characteristics, rejection sensitivity as well as childhood maltreatment. They found that loneliness is highly prevalent in PDD and BPD patients and contributes to the overall symptom burden. In addition, loneliness showed an association with prior experiences of childhood maltreatment as well as current rejection sensitivity. The experience of childhood maltreatment makes PDD patients sensitive to rejection, while their avoidant/submissive interpersonal behavior makes them prone for rejection (Struck et al.) and leads to reduced social connectedness and compassion toward close others (Frick et al.). This cycle needs to be broken.

In CBASP, the focus is on breaking this cycle. Besides individual psychotherapy, CBASP can also be effective in a group format for inpatients and outpatients. For example, Guhn et al. investigated interpersonal change during a multimodal inpatient treatment and found that the majority of their PDD patients reported gain in social competence throughout the CBASP group sessions. In line with these findings is the study by Sürig et al. They examined change in interpersonal and metacognitive skills during treatment with CBASP and metacognitive therapy. They found that especially changes in interpersonal skills seem to be of particular relevance in the treatment of depression. In addition, increases in friendly-dominant behaviors and a less preoperational style of thinking were associated with alleviation of depressive symptoms, thereby, again, supporting McCullough's interpersonal model of depression.

Beside the positive effects of treatment, it is also important to study the negative effects that treatments might have. Herzog et al. focused on the impact of negative effects in a multimodal inpatient CBASP treatment program. They found that most reported negative effects, such as stigmatization, financial concerns and intrapersonal changes, do not appear to have an impact on treatment outcome. However, dependence on the therapist, which was the most frequently reported negative effect, did seem to be negatively linked to treatment response. Glanert et al. also found that care dependency might be associated with a worse treatment outcome in depressed patients. In addition, their results indicate that care dependency is a dynamic construct, as it is changing over time, while the levels of care dependency seem to be independent from the received type of treatment. Future research should continue investigating the mechanisms of care dependency.

Besides PDD, most patients report comorbid disorders, such as comorbid personality disorders. In contrast to other personality disorders, comorbid borderline personality disorder (BPD) is often regarded as an exclusion criterion for CBASP. In clinical settings, however, subthreshold BPD symptoms are prevalent in PDD and may not be obvious at an initial assessment prior to therapy. As data on their impact on CBASP outcome are very limited, this naturalistic study by Konvalin et al. investigates BPD features in PDD and their relevance for the therapeutic outcome of a multimodal CBASP inpatient program. The results show that BPD features at baseline did not limit the clinical response to CBASP. In line with these findings that CBASP might also be useful in patients with other symptom profiles, Sayegh et al. found that patients with bipolar disorder who are currently in a depressive episode can also benefit from CBASP in a group format for outpatients. Finally, Serbanescu et al. investigated the impact of baseline characteristics on the effectiveness of psychotherapy in PDD patients. They compared CBASP with Supportive Psychotherapy and found for both therapies that a poor response was predicted by higher scores of depressive symptoms, suicidality, anxiety, social inhibition, a history of moderate-to-severe emotional or sexual abuse and prior inpatient treatment. In terms of moderators, CBASP was superior over Supportive Psychotherapy for patients with higher depression scores, for patients who had no recurrent depressive episode without complete remission between the episodes, for patients with comorbid axis-I disorders, for patients with a history of at least one antidepressant treatment and for patients with early trauma in the form of moderate-to-severe emotional or physical neglect.

For John, his CBASP treatment taught him how to express negative feelings and how to handle difficult situations with his son, while staying connected with his environment. He knows that he has to keep on working on this, because the patterns that cause his depression are deeply anchored in him.

## Author Contributions

JW wrote the editorial. PK, ES, TFu, and TFa commented on it and agreed with the final version.

## Conflict of Interest

The authors declare that the research was conducted in the absence of any commercial or financial relationships that could be construed as a potential conflict of interest.

## Publisher's Note

All claims expressed in this article are solely those of the authors and do not necessarily represent those of their affiliated organizations, or those of the publisher, the editors and the reviewers. Any product that may be evaluated in this article, or claim that may be made by its manufacturer, is not guaranteed or endorsed by the publisher.

